# Duration of Thyroid Dysfunction Correlates with All-Cause Mortality. The OPENTHYRO Register Cohort

**DOI:** 10.1371/journal.pone.0110437

**Published:** 2014-10-23

**Authors:** Anne Sofie Laulund, Mads Nybo, Thomas Heiberg Brix, Bo Abrahamsen, Henrik Løvendahl Jørgensen, Laszlo Hegedüs

**Affiliations:** 1 Department of Endocrinology and Metabolism, Odense University Hospital, Odense, Denmark; 2 Department of Clinical Biochemistry and Pharmacology, Odense University Hospital, Odense, Denmark; 3 Odense Patient Data Explorative Network OPEN, University of Southern Denmark, Odense, Denmark; 4 Institute of Clinical Research, Odense, Denmark; 5 Research Centre for Ageing and Osteoporosis, Department of Medicine M, Glostrup Hospital, Copenhagen, Denmark; 6 Department of Clinical Biochemistry, Bispebjerg Hospital, Copenhagen, Denmark; Institute of Endocrinology and Metabolism, Islamic Republic of Iran

## Abstract

**Introduction and Aim:**

The association between thyroid dysfunction and mortality is controversial. Moreover, the impact of duration of thyroid dysfunction is unclarified. Our aim was to investigate the correlation between biochemically assessed thyroid function as well as dysfunction duration and mortality.

**Methods:**

Register-based follow-up study of 239,768 individuals with a serum TSH measurement from hospitals and/or general practice in Funen, Denmark. Measurements were performed at a single laboratory from January 1st 1995 to January 1st 2011. Cox regression was used for mortality analyses and Charlson Comorbidity Index (CCI) was used as comorbidity score.

**Results:**

Hazard ratios (HR) with 95% confidence intervals (CI) for mortality with decreased (<0.3 mIU/L) or elevated (>4.0 mIU/L) levels of TSH were 2.22; 2.14–2.30; P<0.0001 and 1.28; 1.22–1.35; P<0.0001, respectively. Adjusting for age, gender, CCI and diagnostic setting attenuated the risk estimates (HR 1.23; 95% CI: 1.19–1.28; P<0.0001, mean follow-up time 7.7 years, and HR 1.07; 95% CI: 1.02–1.13; P = 0.004, mean follow-up time 7.2 years) for decreased and elevated values of TSH, respectively. Mortality risk increased by a factor 1.09; 95% CI: 1.08–1.10; P<0.0001 or by a factor 1.03; 95% CI: 1.02–1.04; P<0.0001 for each six months a patient suffered from decreased or elevated TSH, respectively. Subdividing according to degree of thyroid dysfunction, overt hyperthyroidism (HR_overt_ 1.12; 95% CI: 1.06–1.19; P<0.0001), subclinical hyperthyroidism (HR_subclinical_ 1.09; 95% CI: 1.02–1.17; P = 0.02) and overt hypothyroidism (HR_overt_ 1.57; 95% CI: 1.34–1.83; P<0.0001), but not subclinical hypothyroidism (HR_subclinical_ 1.03; 95% CI: 0.97–1.09; P = 0.4) were associated with increased mortality.

**Conclusions and Relevance:**

In a large-scale, population-based cohort with long-term follow-up (median 7.4 years), overt and subclinical hyperthyroidism and overt but not subclinical hypothyroidism were associated with increased mortality. Excess mortality with increasing duration of decreased or elevated serum TSH suggests the importance of timely intervention in individuals with thyroid dysfunction.

## Introduction

The thyroid hormones thyroxine (T_4_) and triiodothyronine (T_3_) are essential in the regulation of a number of vital pathways such as lipolysis, gluconeogenesis and oxidative phosphorylation [1]. Thyroid dysfunction, as reflected by decreased as well as elevated TSH, has been linked with an increased risk of developing a number of clinical conditions, such as cardiovascular diseases [2], [3], diabetes [2]–[5], various malignant conditions [6], lung diseases [7], and psychiatric diseases [8], [9] – both before and after the diagnosis of thyroid dysfunction. Whether this increased burden of morbidity also manifests as increased mortality in patients with thyroid dysfunction is controversial. During the past decade, several meta-analyses [10]–[15] have dealt with the possible influence of thyroid dysfunction on mortality, but due to considerable heterogeneity in terms of design (definition and severity of thyroid dysfunction, inclusion and exclusion criteria, treatment type and number of individuals included and inconsistent control for comorbidity) it is not possible to draw any firm conclusions. Recently, two large-scale register-based studies from Denmark, where comorbidity was controlled for in a standardized manner, have shown an increased mortality in overt hypo- and hyperthyroidism [16], [17]. However, neither study provided information on biochemical thyroid data permitting an evaluation of the influence of subclinical thyroid dysfunction. Nor did these studies take the duration of thyroid dysfunction into consideration. These shortcomings constitute essential limitations when investigating the consequences of thyroid dysfunction.

Thus, the objective of the present long-term, adequately powered follow-up study was to investigate the consequences of biochemically assessed thyroid dysfunction, as evaluated by a single first measurement of serum TSH and duration of thyroid dysfunction with respect to mortality. The study covers the whole spectrum of thyroid dysfunction from subclinical to overt hypo- and hyperthyroidism, including account of comorbidity and whether the test was obtained in a hospital setting or at a general practitioner (GP).

## Subjects and Methods

### Data sources

The Danish Civil Registration System (DCRS) provides all Danish residents with a unique social security number. The DCRS contains e.g. personal information on birthday, year, gender, place of birth, hospital records and citizenship(s) [18]. The social security number allows for individual record linkage between all Danish health registers [19], [20]. The biochemical data is stored in the OPENTHYRO database, which retrieved information on blood samples analyzed at the Department of Clinical Biochemistry and Pharmacology at Odense University Hospital, including all tests ordered by GPs on the island of Funen. All biochemical data were linked to social security numbers and cross-linked with the National Patient Registry (DNPR) and the National Database of Reimbursed Prescriptions (DNDRP), which contain information about comorbidity from all somatic hospital admissions (both in- and outpatient contacts) [21] and records of all medications dispensed from Danish pharmacies, respectively [22]. Patient information was anonymized and de-identified prior to analysis.

### Study subjects

The initial extraction from OPENTHYRO includes all subjects with at least one TSH measurement between January 1^st^ 1995 and January 1^st^ 2011 (n = 275467). In order to investigate only incident cases a washout period of one year (1995) was used (excluding 5644 subjects). Patients who before the initial TSH measurement had received radioactive iodine, had had thyroid surgery, had received treatment with anti-thyroid medication (carbimazole, propylthiouracil or methimazole) or thyroid hormone, were excluded (n = 8819); as were patients diagnosed with any pituitary disease prior to the initial TSH measurement (n = 753). Patients under the age of 18 years (n = 20260) and those lost to follow-up or emigration were also excluded, leaving 239768 subjects.

### Thyroid function phenotypes

For dose-response analyses by severity of thyroid dysfunction, serum TSH was divided into subgroups. The index date and the thyroid function phenotypes were based on the first serum TSH measurement. Euthyroidism was defined as a TSH value between 0.3–4.0 mlU/L. Decreased TSH covers subjects with a TSH below 0.3 mIU/L. Overt hyperthyroidism was defined as TSH<0.3 mlU/L and T_4_>135.0 nmol/L or T_3_>2.2 nmol/L; subclinical hyperthyroidism as a TSH<0.3 mlU/L and T_4_<135.0 nmol/L and T_3_<2.2 nmol/L. Elevated TSH covers subjects with a TSH above 4.0 mIU/L. Overt hypothyroidism was defined as a TSH>4.0 mlU/L and a T_4_<60.0 nmol/L and, finally, subclinical hypothyroidism was defined as a TSH>4.0 mlU/L and a normal T_4_ (60.0–135.0 nmol/L).

### Assays

All serum TSH determinations were done in the same laboratory, which served all hospitals and general practitioners in the region. Until 2006, TSH values were measured using a time-resolved fluoroimmunoassay based on a direct sandwich technique with three mouse anti-human monoclonal antibodies using the AutoDELFIA equipment (Wallac, Turku, Finland). The limit of detection (LOD) was 0.005 mIU/L, while within-run imprecision had a coefficient of variation (CV) of 2.7% at 0.89 mIU/L and 1.3% at 17.6 mIU/L [23]. From 2006, measurements were performed with a solid-phase, two-site chemiluminescent immunometric assay on Immulite 2000 equipment (Siemens, Erlangen, Germany). The analytical LOD was 0.004 mIU/L. Within-run CV was 5.3% at 0.32 mIU/L and 3.9% at 3.3 mIU/L. To assure compatibility between the two analyses, method comparison was performed in 120 patient samples showing comparable means (range 0.008–49 mIU/L) and a regression coefficient of 0.991. In addition, an external quality control program assured comparability (Ringversuche, RfB, Bonn, Germany).

Concentrations of T_4_ and T_3_ were analyzed using time-resolved fluoroimmunoassays based on competitive binding to T_3_- or T_4_-specific antibodies, respectively. Analyses were performed using the AutoDELFIA equipment (Wallac, Turku, Finland). LOD was 0.3 nmol/L (T_3_) and 5.0 nmol/L (T_4_). CV was 3.2% at 1.37 nmol/L (T_3_) and 2.8% at 79.0 nmol/L (T_4_).

### Morbidity

The Charlson Comorbidity Index (CCI) is based on 19 comorbid conditions (myocardial infarction, congestive heart failure, peripheral vascular disease, cerebrovascular disease, dementia, chronic pulmonary disease, connective tissue disease, ulcer disease, diabetes, hemiplegia, moderate or severe renal disease, diabetes with end organ damage, any tumor, leukemia, lymphoma, mild liver disease, moderate or severe liver disease, metastatic solid tumor and AIDS) [24]. All the disease categories were identified from DNPR. The CCI was applied to hospital contacts in the time period from January 1^st^ 1977 (establishment of DNPR) until end of the study (November 30^th^ 2012).

### Approvals

The project is approved by the Danish Data Protection Agency and by Statistics Denmark to access health records. OPEN is an approved research institution permitted to access data hosted by Statistics Denmark (project 704047).

### Statistics

Data processing was done in SPSS version 19.0 (2010, IBM Corp. IBM SPSS Statistics for Windows, Armonk, NY, USA) and SAS version 9.3 (2011, SAS Institute Inc., Cary, NC, USA) through VPN access to Statistics Denmark.

The primary outcome was mortality. Differences between baseline values of the outcome groups were analyzed using parametric or categorical statistical methods depending on the nature of the data.

Multivariate Cox proportional hazards models were used to compute hazard ratios (HR) for all-cause mortality. HRs were adjusted for age, gender, CCI and treatment setting (general practice or hospital clinic). The validity of the proportional hazards assumption was evaluated by inspection of Schoenfeld residuals versus time. No significant associations between the residuals and time were present. Significant differences between the groups were defined as a P-value below 0.05 using two-tailed tests. In addition, we performed a sub-analysis, where TSH was used as a continuous variable in individuals with TSH within the normal range. A dynamic model was developed to encompass changes in thyroid dysfunction over time. A time-dependent cumulative covariate to capture the number of six month periods during which the mean TSH level had been decreased or elevated, was designed. This took into account all episodes of abnormal TSH in both study groups rather than the baseline classification alone. Any six month periods in which no further TSH measurement had been performed were considered periods of normal TSH for the purpose of this conservative sub-analysis.

## Results

### Baseline characteristics

A total of 239768 subjects (56.3% female and 43.7% male) with at least one serum TSH measurement were identified from the OPENTHYRO database (Table 1). Of the study population, 3.5% were hypothyroid, 92.7% were euthyroid, and 3.8% were hyperthyroid. After subdivision according to thyroid dysfunction phenotype, we found 2.0% of the cohort to be overtly hyperthyroid, 1.0% to be subclinically hyperthyroid, 0.2% to be overtly hypothyroid and 2.7% to be subclinically hypothyroid.

**Table 1:** Baseline population characteristics.

	Decreased TSH			Normal TSH	Elevated TSH		
	Overall	Subclinical	Overt		Overall	Subclinical	Overt
**N**	9217	2408	4857	222138	8413	6519	539
**Age at first TSH** in years (SD)	61.7 (18.1)**	64.0 (17.2)**	58.5 (18.2)**	50.8 (18.1)**	53.6 (19.0)**	54.9 (18.2), NS	58.6 (17.6), NS
**Females** (%)	71.9	65.0	75.6	55.1	71.6	70.9	70.1
**GP/hospital** (%)	35.5/64.5**	42.6/57.4**	39.4/60.6**	45.0/55.0**	42.4/57.6**	43.7/56.3, P = 0.03	36.2/63.8**
**CCI** before first TSH (SD)	0.88 (1.47)**	0.93 (1.49)**	0.68 (1.24)**	0.60 (1.18)**	0.69 (1.34)**	0.65 (1.28)**	0.98 (1.85)**
**CCI** after first TSH (SD)	1.36 (2.01)**	1.40 (2.04)**	1.17 (1.89)**	0.87 (1.70)**	0.98 (1.78)**	0.96 (1.76)**	1.22 (1.96)**
**Deaths** (%)	33.6**	33.6**	25.0**	15.0**	18.5**	18.0**	29.9**
**Median follow-up** in years (SD)	7.7 (4.4)**	7.3 (4.2)**	8.1 (4.1)**	7.5 (3.7)**	7.2 (3.9)**	7.2 (3.8)**	7.1 (4.5)**

**P<0.0001, NS =  not significant, GP =  general practitioner, CCI =  Charlson Comorbidity Index.

Data shown are mean (standard deviation) values, except for percentages. Differences in continuous variables between the group with elevated and decreased serum TSH, respectively, and the group with normal TSH values were tested with independent sample t-tests, whereas differences between categorical variables were tested with chi^2^-tests.

Demographic characteristics are summarized in table 1. The mean age was significantly higher in the group with decreased TSH (61.7 years) when compared with the euthyroid (50.8 years) and the hypothyroid groups (53.6 years). The hyperthyroid individuals were more likely to have a higher burden of comorbidity as reflected by a higher mean CCI (0.88; SD 1.47). Diagnostic setting influenced the burden of comorbidity, as individuals with samples from GP had a mean CCI of 0.37 (SD 0.82), while individuals diagnosed in a hospital setting had 0.81 (SD 1.41), P = 0.0001. During a median follow-up time of 7.4 years, 37964 subjects (18.5% of those with elevated TSH, 15.0% of the normal TSH group and 33.6% of those with decreased TSH) died. Within the group with decreased TSH, mortality was significantly higher for males than for females.

Looking at mortality for the group with high serum TSH, males and females separately, we found a HR 1.20; 95% Cl: 1.11–1.31; P<0.0001) in the male population, after adjusting for the previously mentioned factors. No significant association was found in the female population with high TSH (HR 1.01; 95% Cl: 0.95–1.08; P = 0.64). When investigating the male population with low TSH, we found a significant association with mortality (HR 1.21; 95% CI: 1.16–1.27; P<0.0001). The association was also found to be significant in the low TSH female group with a HR 1.25; 95% CI: 1.17–1.34; P<0.0001.

### Mortality in individuals with decreased TSH versus euthyroid individuals

**Figure 1:**
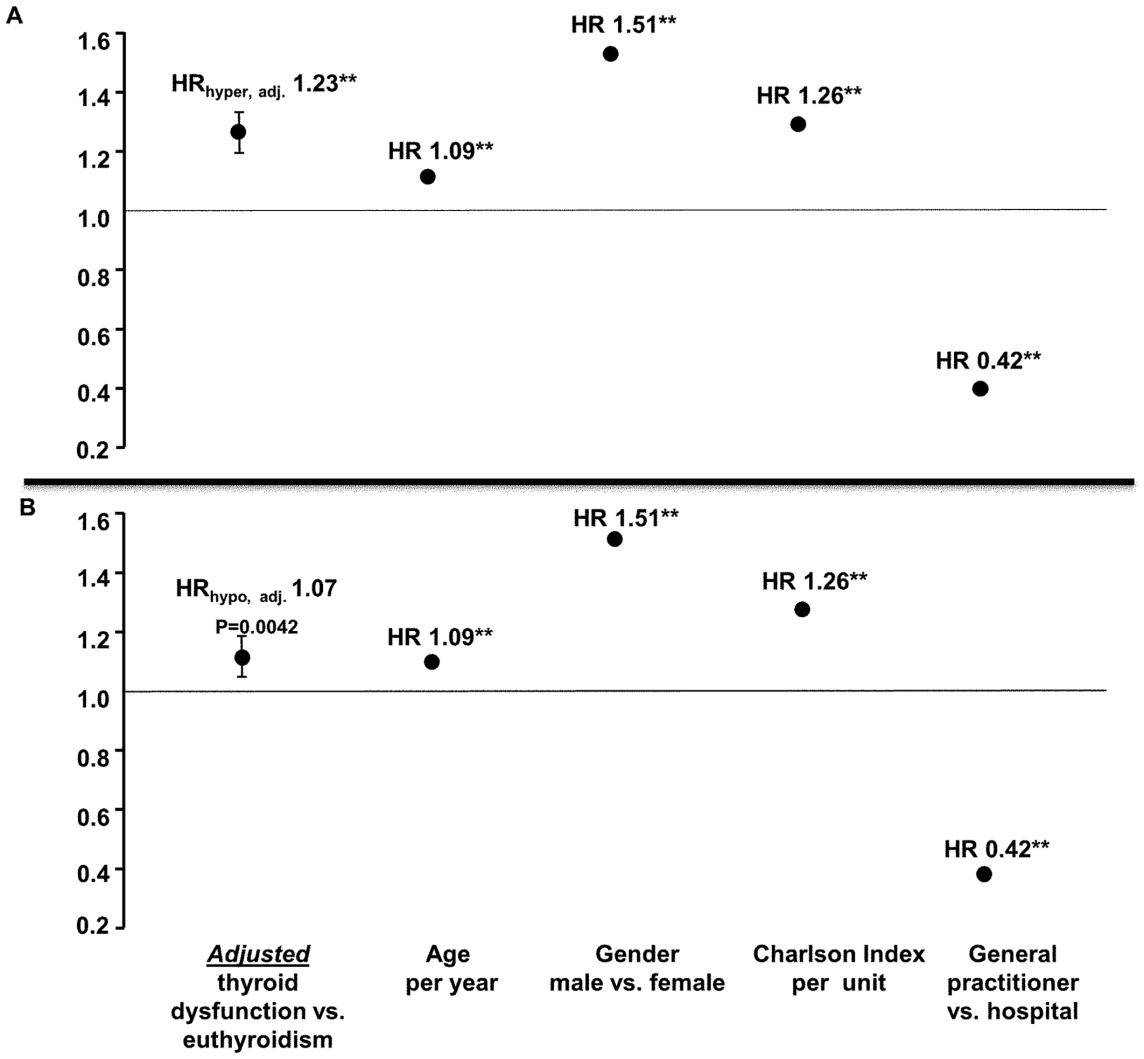
Cox proportional hazards models. Decreased TSH (<0.3 mlU/L) (panel A) and elevated TSH (>4 mlU/L) (panel B) as predictors of mortality. The figure illustrates the crude hazard ratios and the adjusted hazard ratios from the Cox regression analysis with stepwise addition of the covariates. Population N = 239768. ** P<0.0001.

The hazard ratio (with 95% confidence interval) for all-cause mortality in subjects with decreased TSH was 2.31; 95% CI: 2.24–2.39; P<0.0001. After adjusting for confounders the HR was HR 1.23; 95% CI: 1.19–1.28; P<0.0001 (Figure 1, panel A).

### Dose-response analysis. Cumulative exposure to decreased TSH

A Cox proportional hazards model in which a time-dependent covariate had been defined was used to capture the cumulative duration of decreased TSH. For each six month time intervals in which the mean serum TSH value was below 0.3 mIU/L, mortality risk increased by a factor 1.09; 95% CI: 1.08–1.10; P<0.0001. This corresponds to a 137% increase in mortality risk per five years with decreased TSH.

### Degree of hyperthyroidism and mortality

Subdividing into overt and subclinical hyperthyroidism showed significantly increased mortality in both conditions (HR_overt_ 1.12; 95% CI: 1.06–1.19; P<0.0001, and HR_subclinical_ 1.09; 95% CI: 1.02–1.17; P = 0.012) compared to euthyroid individuals (Table 2).

**Table 2:** Unadjusted and adjusted (age, gender, Charlson Comorbidity Index and location of TSH collection) hazard ratios for mortality according to biochemically defined thyroid phenotype with 95% confidence intervals.

	Overt hyperthyroidism vs. euthyroidism	Subclinical hyperthyroidism vs. euthyroidism	Overt hypothyroidism vs. euthyroidism	Subclinical hypothyroidism vs. euthyroidism
**HR_unadj_**	1.56 (1.48–1.66)**	2.31 (2.15–2.48)**	2.12 (1.81–2.47)**	1.25 (1.18–1.33)**
**HR_adj_**	1.12 (1.06–1.19)**	1.09 (1.02–1.17), P = 0.012	1.57 (1.34–1.83)**	1.03 (0.97–1.09), NS

** P<0.0001, NS =  not significant.

### Impact of diagnostic setting (GP or hospital)

Individuals with decreased TSH diagnosed in GP as well as individuals diagnosed in a hospital setting had an increased HR for all-cause mortality (Table 3). The association persisted for individuals with decreased TSH and without comorbidities, whether diagnosed in GP or in a hospital (HR_GP, CCI = 0_ 1.31; 95% CI: 1.17–1.48; P<0.0001, and HR_hospital, CCI = 0_ 1.20; 95% CI: 1.12–1.29; P<0.0001).

**Table 3:** Impact of diagnostic setting (GP or hospital) on mortality risk (hazard ratio) with 95% confidence intervals.

	General Practitioner (N = 6840)		Hospital (N = 15638)	
	Hyperthyroidism vs. euthyroidism	Hypothyroidism vs. euthyroidism	Hyperthyroidism vs. euthyroidism	Hypothyroidism vs. euthyroidism
**HR_adj_**	1.25 (1.16–1.36)** (N = 3275)	1.01 (0.90–1.14), NS (N = 3565)	1.22 (1.17–1.27)** (N = 5942)	1.09 (1.03–1.15), P = 0.0028 (N = 4848)
**HR_adj_, _CS = 0_**	1.31 (1.17–1.48)** (N = 2247)	0.94 (0.80–1.11), NS (N = 2755)	1.20 (1.12–1.29)** (N = 3076)	1.00 (0.90–1.10), NS (N = 2761)

	Subclinical	Overt	Subclinical	Overt	Subclinical	Overt	Subclinical	Overt
**HR_adj_**	1.28 (1.12–1.46), P = 0.0003 (N = 1026)	1.16 (1.04–1.31)** (N = 1914)	0.98 (0.85–1.11), NS (N = 2847)	1.32 (0.90–1.94), NS (N = 195)	1.03 (0.95–1.12), NS (N = 1382)	1.09 (1.02–1.17), P = 0.011 (N = 2943)	1.04 (0.97–1.11), NS (N = 3072)	1.60 (1.35–1.90)** (N = 344)
**HR_adj_, _CS = 0_**	1.31 (1.08–1.60), P = 0.0063 (N = 672)	1.23 (1.05–1.43), P = 0.0095 (N = 1375)	0.90 (0.74–1.08), NS (N = 2195)	1.32 (0.80–2.20), NS (N = 148)	1.11 (0.97–1.28), NS (N = 672)	1.16 (1.05–1.29), P = 0.0049 (N = 1738)	0.98 (0.88–1.10), NS (N = 2114)	1.20 (0.88–1.63), NS (N = 183)

**P<0.0001, NS =  not significant.

After subdividing the samples collected from GP according to biochemical thyroid phenotypes, the association with excess mortality persisted (HR_overt_ 1.16; 95% CI: 1.04–1.31; P<0.0001, and HR_subclinical_ 1.28; 95% CI: 1.12–1.46; P = 0.0003). Essentially, similar results were found among individuals without comorbidity (HR_overt, CCI = 0_ 1.23; 95% CI: 1.05–1.43; P = 0.01, and HR_subclinical CCI = 0,_ 1.31; 95% CI: 1.08–1.60; P = 0.006). In the hospital setting, only overt hyperthyroidism was associated with a statistically significant excess mortality in both the overall group and in the group with no registered comorbidities (HR_overt_ 1.09; 95% CI: 1.02–1.17; P = 0.01, and HR_overt, CCI = 0_ 1.16; 95% CI: 1.05–1.29; P = 0.005). In contrast, subclinical hyperthyroidism diagnosed in a hospital setting was not found associated with excess mortality compared to euthyroid individuals (HR_subclinical_ 1.03; 95% CI: 0.95–1.12; P = 0.5, and HR_subclinical, CCI = 0_ 1.11; 95% CI: 0.97–1.28; P = 0.1).

### Mortality in individuals with elevated TSH versus euthyroid individuals

As evident from figure 1, panel B, subjects with elevated TSH had an increased mortality. The HR for all-cause mortality in subjects with elevated TSH was 1.08; 95% CI: 1.04–1.12; P<0.0001, in the unadjusted setting. After adjusting for confounders, the HR was 1.07; 95% CI: 1.03–1.11; P = 0.001.

### Dose-response analysis. Cumulative exposure to elevated TSH

For each six month time interval in which the mean TSH value was above 4.0 mIU/L, mortality risk increased by a factor 1.03; 95% CI: 1.02–1.04; P<0.0001. This corresponds to a 34% increase in mortality risk per five years with elevated TSH.

### Degree of hypothyroidism and mortality

Subdividing hypothyroidism into overt and subclinical hypothyroidism showed excess mortality in the overtly (HR_overt_ 1.57; 95% CI: 1.34–1.83; P<0.0001), but not in the subclinically hypothyroid population (HR_subclinical_ 1.03; 95% CI: 0.97–1.09; NS) (Table 2).

### Impact of diagnostic setting (GP or hospital)

Subjects diagnosed with elevated TSH in GP did not have a statistically significant excess mortality (HR_GP_ 1.01; 95% CI: 0.90–1.14; NS). In contrast, subjects diagnosed in a hospital setting had an increased risk of all-cause mortality (HR_hospital_ 1.09; 95% CI: 1.03–1.15; P = 0.003), which disappeared completely when looking at the population without comorbidities (HR_hospital, CCI = 0_ 1.00; 95% CI: 0.90–1.10; NS).

In the hospital setting we observed a significant excess mortality for the group with overt hypothyroidism (HR_overt_ 1.60; 95% CI: 1.35–1.90; P<0.0001). However, this association disappeared when looking at the population with no registered comorbidities (HR 1.20; 95% CI: 0.88–1.63; NS). In GP neither subclinical (HR_subclinical, CCI = 0_ 0.90; 95% CI: 0.74–1.08; NS) nor overt hypothyroidism (HR_overt, CCI = 0_ 1.32; 95% CI: 0.80–2.20; NS) was associated with excess mortality (Table 3).

## Discussion

In the following, we will discuss the association between abnormal serum TSH, the duration of this, and all-cause mortality. Furthermore, we will argue that there are separate effects of the biochemically defined four thyroid phenotypes on mortality. Finally, that whether the serum TSH abnormality was diagnosed in general practice or in a hospital setting matters.

### Hyperthyroidism, duration and mortality

In a register-based health care study with biochemical data from 239768 subjects without previously known thyroid disease, we demonstrate that mortality is significantly increased in subjects with a single first TSH measurement below the reference range.

Intuitively, comorbidity has an impact on all-cause mortality, but may also facilitate the development of decreased TSH, attributable e.g. to treatment with iodine-containing drugs and/or non-thyroidal illness. Therefore, it is essential to assess the burden and timing of comorbidity before estimating the independent consequence of a decreased TSH on mortality. Previous studies are inconsistent in respect to comorbidity adjustment. Some have adjusted for cardiovascular diseases [2], [3], some for cancer [6] and one study accounted for comorbidity from a population-based healthcare register [17]. Similar to the latter study by Brandt and coworkers, we identified comorbidity from a health-related register and thus adjusted for a wide range of comorbidities. When using this approach, the adjusted mortality risk was increased by 23%, which is of the same magnitude as demonstrated in previous studies [2], [6], [12], [13], [17], [25]–[27]. In accordance with our previous study [17], subjects with hyperthyroidism and without comorbidity and irrespectively of diagnostic setting had an increased all-cause mortality, suggesting that decreased TSH *per se* is associated with excess mortality, independent of preexisting comorbidity.

Besides replicating the previous findings, we performed a cumulated dose-dependent mortality analysis. For each six months during which the mean TSH was decreased, mortality risk increased by 9%. This corresponds to a 137% increase in mortality risk per five years with decreased TSH, even after adjusting for confounders. Exact details concerning the timing of onset of disease are unattainable to us, but by using the index TSH measurement, and by only including incident cases, we sought to limit this issue. Our findings emphasize the need for timely and efficient intervention in order to minimize the duration of exposure to abnormal TSH.

### Degree of hyperthyroidism and mortality

Previous studies found ambiguous results of an association between subclinical hyperthyroidism and mortality [10], [28]–[30]. Here, we demonstrate a 9% excess mortality associated with subclinical hyperthyroidism. However, this estimate is influenced by the diagnostic setting as patients in GP had a 28% excess mortality compared to euthyroid individuals. On the other hand, despite a higher CCI in a hospital setting, subjects with subclinical hyperthyroidism did not have significant excess mortality (HR 1.03; 95% CI 0.95–1.12; NS). Whether this is due to a more aggressive treatment in a hospital setting compared to a GP setting is unaccounted for. The excess mortality associated with hyperthyroidism has been attributed to systemic effects of thyroid hormones such as cardiac function changes [4], [31], [32] or arrhythmias [33]. This issue has not been addressed in the present work, since data on such systemic changes were not accessible.

Intuitively, more pronounced hyperthyroidism as seen in the overt phenotype should be linked with a poorer prognosis and more severe clinical manifestations, at least when left untreated [34]–[37]. In the present study we found an adjusted 12% and 9% mortality increase in the overtly and subclinically hyperthyroid group, respectively. Based on these findings, our results are in line with a dose-response relationship between thyroid dysfunction and mortality.

### Hypothyroidism, duration and mortality

Considerable heterogeneity with respect for comorbidity, is evident between the studies addressing whether there is a link between elevated TSH and mortality [3], [5], [6], [11], [38]–[44]. In our study, after adjusting for comorbidity, we found a 7% excess mortality in those with a first TSH measurement above the reference range. Patients diagnosed in a hospital setting, as compared to GP, had a slightly poorer prognosis (HR 1.09; 95% CI 1.03–1.15; P = 0.0028). We speculate that this may be due to additional unmeasured comorbidities or frailty [16], [45]. Accordingly, excess mortality was not present in the population without comorbidity (HR 1.00; 95% CI: 0.90–1.10; NS), which is in line with that found in another large scale survey [3].

Our cumulated dose-dependent mortality analysis resulted in a 34% increase in mortality risk per 5 years with elevated TSH, even after adjusting for confounders. Similarly to the results from the hyperthyroid dose-dependent mortality analysis, this result favors early and efficient correction of thyroid dysfunction to reduce all-cause mortality risk.

### Degree of hypothyroidism and mortality

We did not find evidence of increased mortality in subjects with subclinical hypothyroidism, which is in accordance with the majority of the meta-analyses within this field [10], [11], [14], [29], [46]. Overt hypothyroidism is a more severe form of hypothyroidism than subclinical hypothyroidism [47]. Accordingly, this phenotype had a considerably poorer prognosis (57% increased mortality) as compared to euthyroidism, even after adjustment for age, gender, CCI and whether the individual was seen in GP or at hospital. Overall, our data from the hypothyroid individuals suggests that the excess mortality is driven by findings in the clinically more exposed hospital population.

Hitherto, the relationship between the severity of hypothyroidism and all-cause mortality has not been systematically assessed [11], [16]. This is due to considerable heterogeneity between the studies addressing overt hypothyroidism and mortality [3], [5], [6], [37]–[44], [48]–[54]. The heterogeneity is based on lack of all-cause mortality data [49], [51]–[54], differences in inclusion criteria and differences in confounder adjustment. Most studies adjusted for age and gender, but some also adjusted for atherogenic risk factors [39], [49] or comorbidities [3], [44]. Furthermore, one study did not differentiate between overt and subclinical disease [48]. Three studies [6], [38], [39] found excess mortality in patients with overt hypothyroidism compared to euthyroid individuals. Seven studies found no excess mortality in patients with overt hypothyroidism [3], [5], [11], [40]–[44]. In the present study no association was found with mortality in the GP setting (HR 1.32; 95% CI: 0.90–1.94; NS), and in the hospital setting only the overall group had an excess mortality (HR 1.60; 95% CI: 1.35–1.90; P = 0.0001). This correlation with mortality resolved in the group without comorbidity.

### Strengths and limitations

The major strengths of our study include high statistical power, standardized and validated procedures for evaluating the degree of comorbidity, the population-based setting and the long period of follow-up. Other strengths include the analysis of TSH measurements in a single laboratory. Although all TSH measurements were collected in a well-defined geographical area (Funen, Denmark) over a long period of time, this is not to be confused with a random population sample, since the initial investigation of thyroid function was most probably done on a clinical indication. This could obscure possible year-long duration of abnormal TSH at the time of detection.

While we adjusted for most of the established risk factors, covariates such as alcohol intake [55], cigarette smoking [56], body mass index [57], [58] and level of education [59], also important risk factors for mortality and thyroid dysfunction, were unavailable to us. Furthermore, the categorization of subjects was based on a single baseline measurement of TSH. This could lead to misclassification in a small number of subjects because single measurements might not reflect a steady biochemical status but may be influenced also by the circadian variability of the hormone and presence of non-thyroidal illness [23]. If this bias (type 1 error) is present it would increase the false positives. However, similar associations were present in the sensitivity analyses where multiple TSH assessments over time were used to classify abnormal TSH states.

In addition, our study did not take into account that some individuals may change from hyper- to hypothyroidism, or *vice versa* after the initial TSH measurement. The direction and magnitude of this potential bias is unknown. Lastly, it was not possible to distinguish the two major clinical etiologies of thyrotoxicosis. The majority are caused by Graves' disease or toxic nodular goiter. Since the two diseases present with considerable epidemiological and clinical differences [60], [61], it is forthright to assume differences in mortality pattern. Nyrienda and coworkers [5] found no association between mortality and Graves' disease, nor did they find a correlation between mortality and toxic nodular goiter. In contrast, Metso and coworkers [25] found an increased relative risk for mortality in toxic nodular goiter patients, but not in patients with Graves' disease, whereas we have recently shown that both Graves' disease and toxic nodular goiter are associated with excess all-cause mortality, at least in a hospital setting [62].

Previous studies on the impact of thyroid dysfunction on mortality are characterized by a number of shortcomings, the most important being lack of statistical power, which complicates and potentially invalidates interpretation of the data [11]. Since it has been estimated that at least 900 participants are needed in a Cox power calculation to demonstrate an HR increase in mortality of 20% in individuals with thyroid dysfunction [16], it is evident that most studies are underpowered. Even with this very large amount of data, our power for subgroup analyses was limited because of the small number of deaths in these subgroups.

In addition, the majority of the previous studies did not have access to biochemical data and hence had to depend on hospital diagnoses and prescriptions of thyroid hormone and/or antithyroid drugs. Importantly, the present study included diagnosed as well as un-diagnosed individuals and combined raw/unprocessed data and national health register data, which allowed for a systematic and comprehensive collection of comorbidity and mortality data. Furthermore, availability of the setting in which the TSH sample was taken allowed us to investigate the prognostic implications of an abnormal TSH level in relation to whether it was obtained by a GP or in a hospital setting. Thus, our results can be generalized far beyond that limited to hospitalized elderly individuals with a high degree of morbidity.

## Conclusions

In this study a single abnormal serum TSH measurement and an increasing duration of decreased or elevated serum TSH were strong risk factors for mortality. When combining the TSH measurement with T_3_ and T_4_ measurements, overt hyper-, subclinical hyper- and overt hypothyroidism were all found to be strong indicators of excess mortality. Taken together, these findings suggest the importance of timely intervention in individuals with thyroid dysfunction.
